# Anthropometric measures of obesity and associated cardiovascular disease risk in the Eastern Caribbean Health Outcomes Research Network (ECHORN) Cohort Study

**DOI:** 10.1186/s12889-021-10399-3

**Published:** 2021-02-25

**Authors:** Saria Hassan, Carol Oladele, Deron Galusha, Oswald Peter Adams, Rohan G. Maharaj, Cruz M. Nazario, Maxine Nunez, Marcella Nunez-Smith, on behalf of the ECHORN Writing Group

**Affiliations:** 1 Department of Medicine, Yale School of Medicine, 100 Church Street South, Suite A200, New Haven, CT 06510 USA; 2 Emory University School of Medicine, Emory Rollins School of Public Health, Atlanta, GA 30319 USA; 3 University of the West Indies, Cave Hill Campus, Bridgetown, Barbados; 4 University of the West Indies, St. Augustine Campus, St. Augustine, Trinidad; 5 Department of Biostatistics and Epidemiology, Graduate School of Public Health, University of Puerto Rico, Medical Sciences Campus, San Juan, Puerto Rico; 6 University of the Virgin Islands, School of Nursing, St. Thomas, US Virgin Islands

## Abstract

**Background:**

Accurately defining obesity using anthropometric measures that best capture obesity-related risk is important for identifying high risk groups for intervention. The purpose of this study is to compare the association of different anthropometric measures of obesity with 10-year cardiovascular disease (CVD) risk in adults in the Eastern Caribbean.

**Methods:**

Data from the Eastern Caribbean Health Outcomes Research Network (ECHORN) Cohort Study (ECS) were analyzed. The ECS is comprised of adults aged 40 and older residing in the US Virgin Islands, Puerto Rico, Barbados, and Trinidad. 10-year CVD risk was calculated using the American Heart Association (ACC/AHA) ASCVD Risk Algorithm and categorized in the following high-risk groups: > 7.5, > 10, and > 20%. Logistic regression was used to examine associations between four anthropometric measures of obesity (BMI, waist circumference, waist-to-hip ratio, waist-to height ratio) and 10-year CVD risk.

**Results:**

Mean age (SD) of participants (*n* = 1617) was 56.6 years (±10.2), 64% were women, 74% were overweight/obese, and 24% had an ASCVD risk score above 10%. Elevated body mass index (BMI, > 30 kg/m^2^) and waist circumference were not associated with CVD risk. Elevated waist-to-hip ratio (WHR, > 0.9 men, > 0.85 women) and elevated waist-to-height ratio (> 0.5) were associated with all three categories of CVD risk. Area under the receiver curve was highest for WHR for each category of CVD risk. Elevated WHR demonstrated odds of 2.39, 2.58, and 3.32 (*p* < 0.0001) for CVD risk of > 7.5, > 10 and > 20% respectively.

**Conclusion:**

Findings suggest that WHR is a better indicator than BMI of obesity-related CVD risk and should be used to target adults in the Caribbean, and of Caribbean-descent, for interventions.

**Supplementary Information:**

The online version contains supplementary material available at 10.1186/s12889-021-10399-3.

## Background

The prevalence of obesity has nearly tripled since the 1970s worldwide, with numbers reaching 650 million people, threatening the health gains achieved over the past several decades [1–3]. Excess weight accounted for 4-million deaths worldwide in 2015, with 70% of those deaths due to cardiovascular disease (CVD) [2]. Obesity is an independent risk factor for cardiovascular disease—it is associated with higher rates of known cardiovascular risk factors (diabetes, hypertension, hyperlipidemia, and metabolic syndrome) and it leads to accelerated atherosclerosis and ventricular remodeling [4]. Given these associations, obesity prevention and management is of paramount importance in reducing the morbidity and mortality associated with it.

Appropriate targeting of obesity-related interventions depends on the accurate measurement of obesity. Different anthropometric measures have been used to define obesity, but body mass index (BMI) is the most common. Several studies have outlined the limitations of BMI, with these limitations being more prominent in non-white racial and ethnic groups where measures of central obesity are more strongly associated with hypertension, diabetes, and cardiovascular disease [5–15]. It is unclear which of these anthropometric measure(s) of obesity (BMI, waist circumference, waist-to-hip ratio, and waist-to-height ratio) best reflects obesity-related risk in a Caribbean population.

We focus on the Caribbean as it is a region in transition that is experiencing an epidemic of non-communicable disease, with greater than 50% of total life years annually lost to cancer, cardiovascular disease, and hypertension [16, 17]. Furthermore, the prevalence of obesity is almost 25% among the general population, and 31% of deaths are attributed to cardiovascular disease [18–20]. In order to effectively and efficiently address the high burden of obesity, we need to target adults of Caribbean-descent at the highest risk of obesity-related disorders. The objective of this study is to identify the anthropometric measures of obesity in adults in the Eastern Caribbean that are associated with the highest risk of obesity-related disorders, namely cardiovascular disease (CVD). We hypothesize that relative central measures of adiposity (waist-to-hip ratio, waist-to-height ratio) will be more closely associated with 10-year cardiovascular disease risk than body mass index (BMI).

## Methods

### Study sample and baseline data collection

**Fig. 1:**
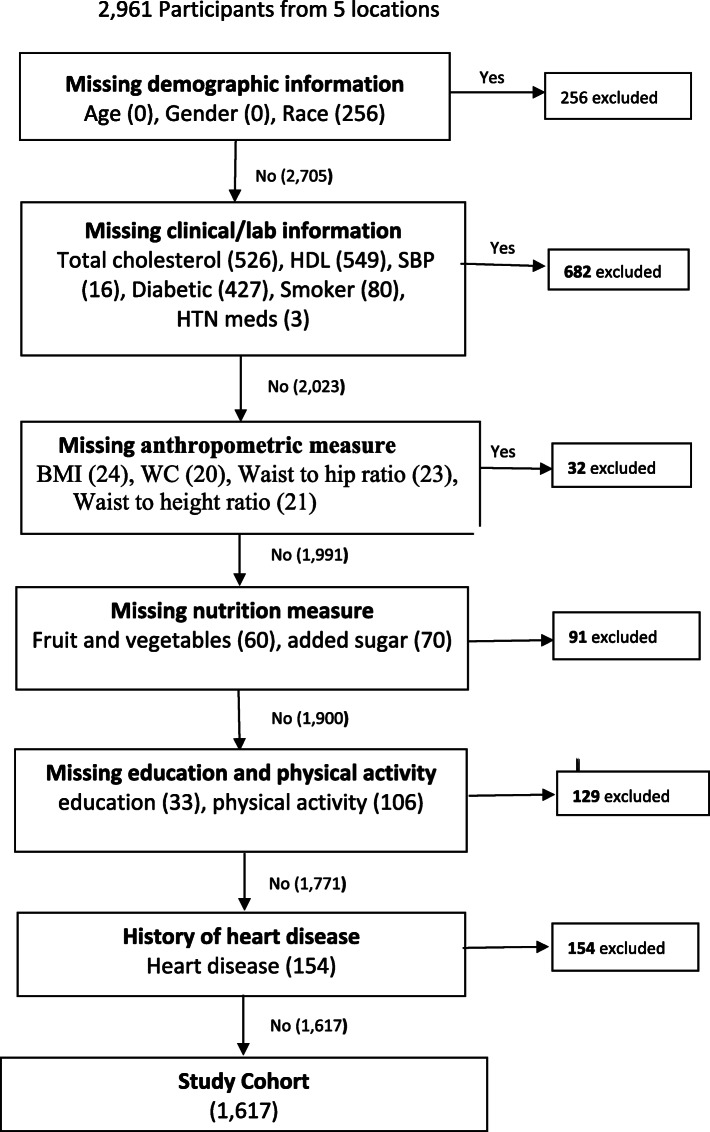
Construction of Study Cohort

This study used baseline data from the Eastern Caribbean Health Outcomes Research Network Cohort Study (ECS). The ECS methodology has been described previously [21–24]. The ECS is an ongoing longitudinal cohort study that follows community-dwelling adults in islands of the U.S. Virgin Islands, Puerto Rico, Trinidad, and Barbados. The ECS study seeks to improve our understanding of risk and protective factors for cardiovascular disease, diabetes, and cancer, in the Eastern Caribbean region [21–24]. Baseline data for the ECS was collected between 2013 and 2018. Participants (*n* = 2961) were recruited on each island using stratified multi-stage random sampling in Barbados, Trinidad, and Puerto Rico, and simple random sampling in the U.S. Virgin Islands of St. Thomas and St. Croix. Eligibility criteria for participation included: English or Spanish speaking, community-dwelling, age 40-years and older. The baseline assessment included a survey (assessing self-reported lifestyle factors, health outcomes, medical history, and demographic characteristics); a clinical assessment (blood pressure, and anthropometric measurements), and laboratory assessment. The survey was developed specifically for the ECS [25]. During data cleaning, outlier values (extremes of values for clinical assessment, laboratory, or survey data) were deemed missing. For the current analysis, we included data for participants with complete anthropometric, laboratory, and medical history data. After removing participants with missing data, our final cohort size was 1617 persons. Figure 1 illustrates how the final sample was derived after removal of missing data.

The ECHORN Cohort Study was approved by the Yale University Human Subjects Investigation Committee, and the Institutional Review Boards of the University of the West Indies, the Ministry of Health Trinidad and Tobago, the University of Puerto Rico, Medical Sciences Campus and the University of the US Virgin Islands. Informed consent was required for participation in the study.

### Measures

#### Main independent variable

Our main independent variable, obesity, was defined by anthropometric measurements taken during the baseline clinical assessment. Trained research nurses conducted waist, hip, height, and weight measurements for all participants. Weight measurements were obtained using a standardized calibrated scale at all sites. Height was determined using the same stadiometer at each site. Waist circumference (WC) and hip circumference were measured using standardized protocols and metric tape measures; measurements were done twice until there was less than a 1cm difference between measurements. Body mass index (BMI) was calculated using the formula: BMI = [weight(kg)/)height(m)^2^]; waist-to-hip ratio (WHR) and waist-to-height ratio (WHtR) were simple ratios of the waist circumference to the hip circumference and waist circumference to height respectively. We dichotomized measurements (elevated vs. not elevated) based on World Health Organization guidelines for inclusion in analyses: elevated BMI defined as ≥30 kg/m^2^; elevated WHR defined as > 0.9 (men), > 0.85 (women); elevated WHtR defined as > 0.5; and WC defined as >40in (men), >35in (women) [11]. In clinical practice, obesity is dichotomized as obese versus non-obese, therefore, dichotomizing each anthropometric measure in our study increases its clinical relevance.

#### CVD Risk

The dependent variable was CVD risk. We estimated CVD risk using the American College of Cardiology/American Heart Association CVD (ASCVD) pooled risk equations [26]. Variables incorporated in the ASCVD calculation include: age, gender, race, smoking status, total cholesterol, systolic blood pressure, diastolic blood pressure, hypertension treatment, and diabetes. Age, gender, race, and smoking status were obtained through participant self-report. Race and ethnicity were determined by answers to the following question: “To which racial or ethnic group or groups would you say you belong? (Check all that apply).” Response options included: white, black or African, Caribbean, Asian (for example Japanese, Chinese, Laotian, Thai, Pakistani or Cambodian), East Indian, Hispanic or Latino, Mixed or multi-racial, Puerto Rican or Boricua, or other. The CVD risk score requires that one designates participants as “African American” or “Other.” Individuals who self-identified as “black or African” or “Caribbean” were classified as “African American” for the calculation. All others were classified as “Other” for the calculation. Smoking status was dichotomized based on self-report to the following question: “Do you still smoke cigarettes, cigars, or tobacco pipe regularly? By regularly we mean at least 20 cigarettes or 1 cigar or half an ounce sachet of loose tobacco per month.” Systolic and diastolic blood pressure were estimated from the mean of two seated blood pressure measurements taken 5 minutes apart during the ECS clinical assessment. Treatment for hypertension was assessed by participant self-reported medication history. Diabetes status was measured using hemoglobin A1C values obtained from laboratory testing (hemoglobin A1c ≥ 6.5% or fasting plasma glucose ≥126 mg/dl) and self-report. We ascertained total cholesterol and high-density lipoprotein (HDL) values from laboratory tests.

We created three categories of 10-year CVD risk (> 7.5, > 10, > 20%) to examine the discriminatory ability of anthropometric measurements to capture various levels of CVD risk. These risk categories indicate that an individual has a greater than 7.5, 10, or 20% chance of atherosclerotic cardiovascular disease (heart attack or stroke) in the next 10 years. Risk categories were determined based on clinical importance and thresholds for therapeutic interventions to reduce CVD risk [27, 28]. Specifically, 7.5% risk is the cut off to determine if individuals with hyperlipidemia warrant statin therapy [29]. A cut off of 10% determines the blood pressure goal for individuals with hypertension and consideration of the use of aspirin for primary prevention of cardiovascular disease [30]. Individuals with greater than 20% risk are deemed high risk and need statin therapy with the goal of reducing their cholesterol by 50% or more [29]. Clinically, the ASCVD risk score is used as a cut off (greater than 7.5, 10, or 20%) and not a range; therefore, the analysis was done using these cut off values as opposed to mutually exclusive ranges.

#### Covariates

Covariates included education level, physical activity, added sugar intake, and fruit and salad/vegetable (FV) intake. Education was measured by self-report and grouped into high school (or secondary school) graduate or less, some college, and college and higher. Physical activity was measured using the WHO Global Physical Activity Questionnaire and was categorized as low, moderate, and high. Added sugar and FV intake were measured by the National Cancer Institute Dietary Screener Questionnaire (DSQ). ECS participants completed the DSQ which included questions that assessed added sugars from beverages and fruit and vegetable intake. Established DSQ scoring algorithms were used to calculate daily teaspoons of added sugar and servings of fresh fruits (e.g, one serving =1 medium mango, 1 large orange, ½ papaya) and vegetables (e.g., one serving = 2 medium carrots, 1 cup leafy greens) [31, 32]. Common demographic risk adjustment variables (e.g., age, race, sex) were included in the CVD risk calculation and were therefore not adjusted for in multivariate models.

#### Analytic methods

We first examined the distribution of variables included in the CVD risk score in our study sample. We then conducted bivariate analyses using chi-squared tests to examine associations between anthropometric measures and levels of CVD risk. We used logistic regression modeling to compare differences in the association between anthropometric measurements (BMI, WHR, and WHtR) and varied levels of CVD risk (> 7.5, > 10, > 20%). WC showed no significant association (*p* > 0.1) with 10-year CVD risk score and therefore was not included in the logistic regression analyses. We ran separate models for each anthropometric measure examined, stratified by gender. Analyses were adjusted for educational level, physical activity, fruit and vegetable intake, and added sugar intake. We secondarily conducted linear regression models for each anthropometric measure to determine if a similar association was observed for a continuous increase in 10-year CVD risk score. To determine and compare the discriminatory ability of anthropometric measures for 10-year CVD risk, we evaluated models using three different indices: Area under the Curve (AROC), Integrated Discrimination Improvement (IDI), and Net Reclassification Index (NRI) [33]. For all results shown, an alpha of 0.05 was used to determine association, where an alpha> 0.05 signifies no association.

## Results

ECS sample characteristics are presented in Table 1. The largest proportion of participants were between the ages of 50–69 years (51%), women (64.3%), and had less than a high school education (30.4%). With regards to the racial and ethnic background of participants, 51.2% self-reported as Black/Caribbean, 7.7% East Indian, 20.7% Hispanic, 12.1% Mixed, and 8.2% White. Thirty-eight percent of the sample had elevated BMI (> 30 kg/m^2^), 81% had elevated blood pressure (> 120 mmHg systolic), and 26% had a history of diabetes. Eight percent of participants reported smoking regularly, 40% had total cholesterol greater than 200 mg/dl, and 71% had an HDL ≤ 60 mg/dl. Thirty-eight percent of participants had BMI > 30 kg/m2, 50% elevated WHR, 48% elevated WC, and 79% elevated WHtR.

**Table 1:** Study Sample Characteristics and Distribution of Measures Used in the ASCVD Risk Calculator

Characteristic	Total (*n* = 1617)
	n (%)
**Age (years)**	
40–49	442 (27.3)
50–59	588 (36.4)
60–69	397 (24.6)
70+	190 (11.8)
**Gender**	
Male	577 (35.7)
Female	1040 (64.3)
**Race**	
Black/Caribbean	828 (51.2)
East Indian	124 (7.7)
Hispanic/PR	335 (20.7)
Mixed	195 (12.1)
Other	3 (0.2)
White	132 (8.2)
**Education level**	
less than high school	491 (30.4)
high school	374 (23.1)
some college	387 (23.9)
college+	365 (22.6)
**Systolic BP (mmHg)**	
< 120	314 (19.4)
120–129	378 (23.4)
130–139	339 (21.0)
140–149	271 (16.8)
150–159	105 (6.5)
160+	210 (13.0)
**Smoking**	
No	1489 (92.1)
Yes	128 (7.9)
**Total cholesterol (mg/dl)**	
< 200	970 (60.0)
200–239	468 (28.9)
240+	179 (11.1)
**HDL (mg/dl)**	
< 40	268 (16.6)
40–60	881 (54.5)
> 60	468 (28.9)
History of diabetes	
No	1204 (74.5)
Yes	413 (25.5)
BMI (kg/m2)	
< 25	417 (25.8)
overweight 25–29.9	587 (36.3)
0bese > =30	613 (37.9)
Waist-to-hip Ratio	
not elevated	803 (49.7)
elevated	814 (50.3)
Waist Circumference	
not elevated	842 (52.1)
elevated	775 (47.9)
Waist to height ratio	
not elevated	338 (20.9)
elevated	1279 (79.1)
Education level	
less than high school	491 (30.4)
high school	374 (23.1)
some college	387 (23.9)
college+	365 (22.6)
Physical Activity	
low	734 (45.4)
moderate	309 (19.1)
high	574 (35.5)
Serving of fruit and vegetables per week - mean (sd)	9.8 (6.5)
Daily added sugar intake (tsp) without cereal - mean (sd)	13.9 (9.9)

We compared the characteristics of individuals from ECS who were included and excluded from the analytic study sample (due to incomplete data) (see Appendix A). We found no difference in gender, proportion with elevated BMI, WHR, or WHtR. There was a difference in the distribution of participants by island site, which also led to a difference in distribution of participants by race (Black/Caribbean in the included sample was 51.2%; in the excluded sample, 66.0%; *p* < 0.002). Lastly, there was a difference in mean age between participants in the sample and those excluded (56.6 vs 58.1, respectively; *p* < 0.0001).

Table 2 shows the distribution of clinical and lifestyle characteristics for each 10-year CVD risk category. Thirty-two percent of participants had a 10-year CVD risk score greater than 7.5, 24% with CVD risk greater than 10, and 10% with CVD risk greater than 20%. Results of chi-squared tests showed that elevated WHR and WHtR ratio are associated with elevated 10-year CVD risk of > 7.5% (*p* < 0.0001, *p* = 0.0115 respectively), > 10% (*p* < 0.0001, *p* = 0.0034 respectively), and > 20% (*p* < 0.0001, *p* = 0.0184, respectively). WC did not show an association in bivariate analyses with any of the 10-year CVD risk categories. BMI showed an association only with CVD risk > 7.5% compared to < 7.5% (*p* = 0.0113), however, a larger proportion of individuals *without* elevated BMI had elevated CVD risk (66.5%) compared to those with elevated BMI (33.5%). Level of physical activity and added sugar intake were associated with 10-year CVD risk score of > 20% (*p* = 0.0052, *p* = 0.0018 respectively) but not the other CVD risk categories. Fruit and vegetable intake were not associated with CVD risk score. Education level was associated with 10-year CVD risk of > 7.5, > 10, and > 20% with *p* < 0.0001.

**Table 2:** Distribution of clinical and lifestyle characteristics for each 10-year CVD risk category

Characteristic	CVD 10-Year Risk								
	≤7.5% (*n* = 1089)	> 7.5% (*n* = 528)	*p*-value*	≤10% (*n* = 1222)	> 10% (*n* = 395)	*p*-value*	≤20% (*n* = 1451)	> 20% (*n* = 166)	*p*-value*
**Elevated BMI - n (%)**			**0.0113**			0.0786			0.8752
not elevated	653 (60)	351 (66.5)		744 (60.9)	260 (65.8)		900 (62)	104 (62.7)	
elevated	436 (40)	177 (33.5)		478 (39.1)	135 (34.2)		551 (38)	62 (37.4)	
**Waist-to-hip Ratio - n (%)**			**<.0001**			**<.0001**			**<.0001**
not elevated	616 (56.6)	187 (35.4)		675 (55.2)	128 (32.4)		762 (52.5)	41 (24.7)	
elevated	473 (43.4)	341 (64.6)		547 (44.8)	267 (67.6)		689 (47.5)	125 (75.3)	
**Waist Circumference - n (%)**			0.1099			0.2804			0.8134
not elevated	552 (50.7)	290 (54.9)		627 (51.3)	215 (54.4)		757 (52.2)	85 (51.2)	
elevated	537 (49.3)	238 (45.1)		595 (48.7)	180 (45.6)		694 (47.8)	81 (48.8)	
**Waist to height ratio - n (%)**			**0.0115**			**0.0034**			**0.0184**
not elevated	247 (22.7)	91 (17.2)		276 (22.6)	62 (15.7)		315 (21.7)	23 (13.9)	
elevated	842 (77.3)	437 (82.8)		946 (77.4)	333 (84.3)		1136 (78.3)	143 (86.1)	
**Education level - n (%)**			**<.0001**			**<.0001**			**<.0001**
less than high school	272 (25)	219 (41.5)		324 (26.5)	167 (42.3)		408 (28.1)	83 (50)	
high school	257 (23.6)	117 (22.2)		291 (23.8)	83 (21)		341 (23.5)	33 (19.9)	
some college	271 (24.9)	116 (22)		299 (24.5)	88 (22.3)		355 (24.5)	32 (19.3)	
college+	289 (26.5)	76 (14.4)		308 (25.2)	57 (14.4)		347 (23.9)	18 (10.8)	
**Physical Activity - n (%)**			0.4951			0.2489			**0.0052**
low	495 (45.5)	239 (45.3)		551 (45.1)	183 (46.3)		646 (44.5)	88 (53)	
moderate	200 (18.4)	109 (20.6)		225 (18.4)	84 (21.3)		271 (18.7)	38 (22.9)	
high	394 (36.2)	180 (34.1)		446 (36.5)	128 (32.4)		534 (36.8)	40 (24.1)	
**Serving of fruit and vegetables per week - mean (sd)**	9.8 (6.6)	9.7 (6.3)	0.9720	9.7 (6.6)	9.8 (6.3)	0.3734	9.7 (6.6)	10.1 (5.9)	0.2490
**Daily added sugar intake (tsp) without cereal - mean (sd)**	14.2 (10.2)	13.6 (9.3)	0.1048	14.2 (10)	13.3 (9.5)	**0.0258**	14.2 (10)	11.9 (8.3)	**0.0018**

Values are n | (col %) for categorical variables. *P-value connotes significance of chi-squared test of association between high and low risk CVD risk category (i.e. >7.5% vs.  < =7.5%, >10% vs.  < =10%, and >20% vs.  < =20%)) for each anthropometric measure or sociodemographic or lifestyle characteristics. Significant values *p* < 0.05 denoted with bold. Elevated BMI defined as ≥30 kg/m2; elevated WHR defined as > 0.9 (men), > 0.85 (women); elevated WHtR defined as > 0.5

*CVD* Cardiovascular disease, *BMI* Body Mass Index, *WHR* Waist-to-hip ratio, *WHtR* waist-to-height ratio

Multivariate logistic regression model results are presented in Table 3, stratified by gender. Overall, results for each 10-year CVD risk category showed that elevated WHR conferred the greatest odds of increased 10-year CVD risk of > 7.5, > 10, and > 20%. For 10-year CVD risk > 7.5%, the odds ratio for WHR was 2.39 (95% CI: 1.92–2.98) compared to 0.72 (95% CI: 0.58–0.91) and 1.41 (95% CI: 1.07–1.86) for BMI and WHtR respectively. We observed the same pattern for other CVD risk categories. Elevated BMI conferred a lower risk of CVD risk > 7.5%, 0.17 (95% CI: 0.58–0.91). Elevated BMI did not confer a statistically significant increased odds of 10-year CVD risk of > 10% or > 20% using an alpha of significance of 0.05. Race-stratified models (African American versus Other) also showed that WHR conferred the greatest odds of increased 10-year CVD risk across categories (Appendix B).

**Table 3:** Odds of elevated cardiovascular risk by elevated BMI, waist-to-hip ratio and waist-to-height ratio

Anthropometric Measure	Odds of CVD Risk > 7.5%		Odds of CVD Risk > 10%		Odds of CVD Risk > 20%	
	OR (95% CI)	*p*-value	OR (95% CI)	*p*-value	OR (95% CI)	*p*-value
	**TOTAL**					
Elevated BMI	0.72 (0.58,0.91)	0.0049	0.78 (0.61,0.99)	0.0433	0.89 (0.63,1.26)	0.5183
Elevated Waist to Hip Ratio	2.39 (1.92,2.98)	<.0001	2.58 (2.02,3.29)	<.0001	3.32 (2.28,4.83)	<.0001
Elevated Waist to Height Ratio	1.41 (1.07,1.86)	0.0143	1.56 (1.14,2.12)	0.0053	1.60 (1.00,2.57)	0.0496
	**MEN**					
Elevated BMI	1.00 (0.69,1.46)	0.9911	0.96 (0.65,1.40)	0.8178	1.25 (0.77,2.03)	0.3655
Elevated Waist to Hip Ratio	2.85 (2.00,4.07)	<.0001	3.16 (2.17,4.58)	<.0001	3.45 (2.07,5.76)	<.0001
Elevated Waist to Height Ratio	1.94 (1.31,2.87)	0.001	2.22 (1.46,3.39)	0.0002	2.86 (1.55,5.27)	0.0008
	**WOMEN**					
Elevated BMI	0.79 (0.58,1.09)	0.1544	0.92 (0.65,1.31)	0.6449	1.10 (0.63,1.92)	0.7263
Elevated Waist to Hip Ratio	2.09 (1.52,2.87)	<.0001	2.08 (1.46,2.96)	<.0001	2.90 (1.58,5.32)	0.0006
Elevated Waist to Height Ratio	1.83 (1.15,2.92)	0.0107	1.73 (1.03,2.89)	0.0385	1.22 (0.55,2.71)	0.6199

Adjusted multivariate regression analysis showing odds ratio for designated 10-year cardiovascular risk score category by anthropometric measure. Adjusted for educational level, added sugar intake, fruit/salad intake and physical activity level. Elevated BMI defined as ≥30 kg/m2; elevated WHR defined as > 0.9 (men), > 0.85 (women); elevated WHtR defined as > 0.5

*CVD* Cardiovascular disease, *OR* odds ratio, *BMI* Body Mass Index, *WHR* Waist-to-hip ratio, *WHtR* waist-to-height ratio

In gender-stratified results, both elevated WHR and WHtR were associated with greater odds of 10-year CVD risk among men compared to women at all CVD risk levels. Elevated WHR conferred a higher odds of elevated CVD risk in men than the model in women: men with elevated WHR had approximately three-times greater odds of CVD risk > 7.5% (2.00–4.07) and > 10% (2.17–4.58) than men with normal WHR. In women, elevated WHR conferred approximately a two-fold increased odds of CVD risk > 7.5% (1.52–2.87) and > 10% (11.46–2.96) compared to women with normal WHR. In gender-stratified results, elevated BMI showed no significant change in odds of elevated CVD risk at > 7.5, > 10, and > 20% levels. In the linear regression analysis, similar associations were observed for each anthropometric measure for a continuous increase in CVD risk score. Here, beta for elevated BMI (vs. non-elevated BMI) was − 0.778 (*p* = 0.1168); beta for elevated WHR (vs. non-elevated WHR) was 4.29 (*p* < 0.0001); beta for elevated WHtR (vs. non-elevated WHtR) was 2.07 (*p* = 0.0005). This corroborates the logistic regression findings.

**Fig. 2:**
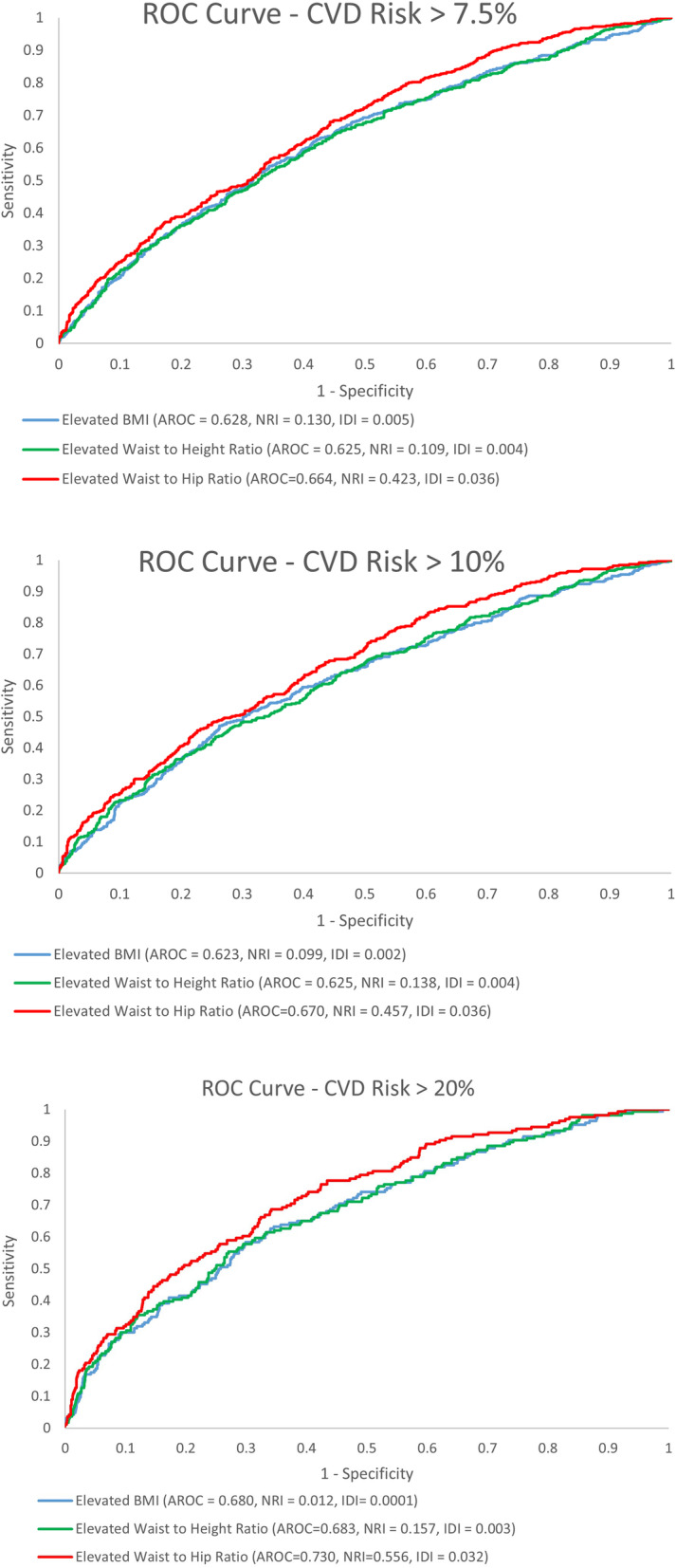
Receiver Operating Characteristic (ROC) curves and model prediction performance indicators for three anthropometric measures of obesity and 10-year CVD Risk. Receiver Operating Characteristic Curve for elevated BMI (blue), elevated waist-to-height ratio (green) and elevated waist-to-hip ratio (red) for 10-year CVD risk score greater than **a** 7.5%, **b** 10% and **c** 20%. AROC = area under the receiver operating curve); NRI = Net Reclassification Index; IDI = integrated Dissemination Improvement. Waist-to-hip ratio performs better across all performance indicators at all levels of 10-year CVD risk

In Fig. 2, we show that model prediction performance indicators consistently demonstrated WHR measurement has better predictive ability for estimating 10-year CVD compared to BMI and WHtR. The ROC curves and the associated AROC, NRI, and IDI values are consistent with multivariate model findings. Figure 2 shows that the discriminatory ability of WHR for elevated 10-year CVD risk score is better across all CVD risk categories. These results are consistent across all 3 performance indicators of AUC, IDI, and NRI. We compared the statistical significance of the difference in AUC for WHR versus WHtR and BMI. For CVD risk > 7.5%, the AUC for WHR was 0.66, compared to WHtR 0.63 (*p* = 0.0005), BMI 0.63 (*p* = 0.0082); for CVD risk> 10%, the AUC for WHR was 0.67 compared to WHtR 0.63 (*p* = 0.0002) and BMI 0.62 (*p* = 0.0013); for CVD risk> 20%, the AUC for WHR was 0.73 compared to WHtR 0.68 (*p* = 0.0005) and BMI 0.068 (*p* = 0.0015).

## Discussion

This study demonstrates that relative central measures of obesity (WHR and WHtR) in adults in the Eastern Caribbean are more strongly associated with cardiovascular risk than BMI. WHR and WHtR confer greater odds of 10-year CVD risk than BMI; importantly, elevated BMI was not associated with increased odds of CVD risk. This suggests that BMI alone should not be used to target high risk individuals for obesity-related screening and interventions among adults in the Eastern Caribbean. Furthermore, WC alone was not associated with increased CVD risk. This is a significant finding given the frequent use of WC as a measure of central adiposity in clinical settings. WHR had greater discriminatory power for 10-year CVD events compared to WHtR and may be a superior measure of high-risk adiposity in this population.

Our study is consistent with previous studies that have indicated that central measures of adiposity are better indicators of obesity-related risk than BMI [5–7, 34–44]. Our study adds to the existing literature by focusing on a Caribbean population, assessing cardiovascular risk using a composite score rather than individual CVD risk factors, and including all anthropometric measures of obesity. Previous studies in the Caribbean have looked only at island-specific populations and showed mixed results with respect to the preferred anthropometric measure [34, 38–41]. The ECS is a unique adult cohort that includes participants from four island sites, allowing for conclusions applicable to a larger group of Eastern Caribbean adults. Furthermore, using the ASCVD risk score increases the significance as a composite score accounts for all significant CVD risk factors – hypertension, diabetes, cholesterol, smoking, and age. The association of anthropometric measures with the composite score provides a more accurate representation of the true discriminatory accuracy of the measures to identify individuals with obesity at highest risk. There have only been a few studies that have looked at this association, and none have been in Caribbean or Caribbean-descent adults [42, 43].

Most prior studies comparing different anthropometric measures of obesity have not examined all three central measures of obesity – WC, WHR, and WHtR [6, 7, 35–37, 44, 45].; this makes it difficult to conclude definitively which measure is optimal. A recent study looking at the population of African American adults in the Jackson Heart Study compared WC, BMI, and WHtR and found WHtR to be more strongly correlated with CVD risk factors (HDL, triglycerides, diabetes, hypertension) compared to BMI [46]. Importantly, however, WHR was not examined. A large meta-analysis by Ashwell et al.*,* corroborated these findings indicating the superiority of WHtR to BMI [47]. However, these studies did not consider WHR. The Hispanic Community Health Study/Study of Latinos (HCHS/SOL) did compare six measures of obesity with cardiometabolic outcomes, including WHR and WHtR. They showed WHR to be associated with increased cardiometabolic condition prevalence compared to other measures [36]. However, this study only looked at independent cardiometabolic outcomes, not a composite CVD risk score. By including both WHtR and WHR in our study, we add to this literature by showing that WHR is superior to WHtR, and demonstrate this more definitively using a composite score of 10-year CVD risk. Importantly, we confirm the decreased utility of WC alone – showing no association of WC with elevated CVD risk. Furthermore, it is noteworthy that similar to prior studies, we show that elevated anthropometric measures of central obesity confer a higher odds of CVD risk and risk factors in men than in women [36, 42]. Nevertheless, our study provides the ability to confirm that the superiority of WHR versus other measures remains consistent across genders in these studies.

The gender stratified results present an opportunity to explore the potential significance of the choice of measure on risk assessment for men versus women. Interestingly, elevated WHR conferred a significantly higher odds of CVD risk at > 7.5, > 10, and > 20% level for both men and women. However, waist-to-height ratio conferred a lower odds of CVD risk for women than in men. Therefore, while WHR can be used as a marker of CVD risk in both men and women, WHtR is likely more useful in men than women. Interestingly, without gender stratification, BMI conferred a lower odds of CVD risk across categories; this does not hold in the gender stratified results and is not significant in the linear regression model.

Our study underscores the high prevalence of obesity in this population (39%) comparable to prevalence estimates in the HCHS/SOL study and the general US population [48, 49]. Furthermore, we provide more evidence for the association of obesity and cardiovascular risk. In this study, individuals with elevated WHR had 2.45 increased odds of having a greater than 10% risk of a major cardiac event in next 10 years than those with a normal WHR. This indicates the urgent need for obesity-related and CVD-risk reduction interventions in this population, starting with accurately identifying those at risk. We make the argument here that BMI alone cannot be used to target Eastern Caribbean adults with obesity for interventions.

There are a few limitations of the study that should be noted. The first is that we use the AHA/ACC pooled ASCVD risk score to determine the risk of a CVD event in 10 years. The pooled risk equations of the ASCVD risk score were not derived from a multi-ethnic or Caribbean population and therefore carries limitations to accuracy in our Eastern Caribbean adult cohort [26]. Prior studies using this risk score have indicated that it may overestimate 10-year CVD risk [50–52]. However, other studies have shown that in black populations it performs adequately and equally as well as the Framingham risk score [53]. While the ASCVD risk calculator may not be an accurate predictor of CVD risk in this population, it is the one most widely available and used. Data from follow up waves of ECS will allow us to assess the accuracy of ASCVD risk score in predicting CVD outcomes in this population. For the current study, the same risk calculator was used across anthropometric measures, therefore, the limitations of the ASCVD algorithm do not affect the association of BMI, WHR, and WHtR with CVD risk that is the purpose of this study. Additionally, in applying the pooled ASCVD equations, we grouped individuals who self-identified as black/Caribbean as “African American,” and everyone else as “Other.” This classification may not be accurate if all individuals of Caribbean-descent indeed have higher risk of CVD comparable to that of African Americans. We conducted a sensitivity analysis to understand the effect of changing how we group ECS participants. If we place all participants except those who self-identify as “white” under “African American” for purposes of the calculation, the strong association of WHR with CVD risk and the lack of such association for BMI and WC remains. These results can be viewed in Appendix C. Another limitation is that our exclusion of participants with missing values may have introduced selection bias. This possibility was raised by there being a difference between the excluded and included groups by island site, race/ethnicity, and age group as indicated in Appendix A. This was likely due to lack of laboratory availability for a limited period in some island sites versus others (affecting the site and race/ethnicity distribution), and the exclusion of individuals with history of heart disease meant the excluded group would likely be older. Another limitation is that of recall bias, given dietary and physical activity were only assessed using questionnaires. Another limitation is that we did not have percentage body fat measurement available for ECS participants. Prior studies have shown that percentage of body fat is the best measure of true adiposity and most closely correlates with mortality and CVD-related risk [54]. However, measuring percentage body fat is not currently routine clinical practice and not feasible in most sites. With results from future waves of ECS, we will be able to determine which anthropometric measures are associated with incident CVD, which would prove more accurate than the CVD risk calculator. We will also be able to compare the accuracy of the various CVD risk calculators and determine which is a more precise reflection of CVD risk in this population.

## Conclusion

Study findings demonstrate the superiority of WHR for identifying adults in the Eastern Caribbean with elevated 10-year CVD risk. These findings, combined with the totality of epidemiological evidence on WHR, WC, and BMI, indicate that WHR should be used in addition to BMI and WC to target individuals for screening, nutrition counseling, and lifestyle and weight-loss interventions. Consideration should be given to include WHR in the routine clinical assessment of Caribbean and Caribbean-descent individuals to ensure that adults with high risk obesity are identified for intervention, enabling health systems to curb the rising burden of obesity-related disorders in the region.

## Supplementary Information

**Additional file 1: Appendices. Appendix A.** Comparison of included and excluded cases from the ECS**. Appendix B.** Odds of elevated cardiovascular risk by elevated BMI, waist-to-hip ratio and waist-to-height ratio stratified by race**. Appendix C.** Odds of elevated cardiovascular risk by elevated BMI, waist-to-hip ratio and waist-to-height ratio [all participants classified as “AFRICAN AMERICAN” except those who self-identify as white – classified as “OTHER” for ASCVD Calculation].

## Data Availability

The datasets used during the current study are available from the corresponding author on reasonable request.

## Abbreviations

AROC: Area under the Curve
ASCVD: American Heart Association (ACC/AHA) cardiovascular disease
BMI: Body mass index
CVD: Cardiovascular disease
DSQ: Dietary screening questionnaire
ECHORN: Eastern Caribbean Health Outcomes Research Network
ECS: ECHORN Cohort Study
IDI: Integrated Discrimination Improvement
NRI: Net Reclassification Index
WC: Waist circumference
WHO: World Health Organization
WHR: Waist-to-hip ratio
WHtR: Waist-to-height ratio

## References

[1] Vos T, Abajobir AA, Abate KH, Abbafati C, Abbas KM, Abd-Allah F (2017). Global, regional, and national incidence, prevalence, and years lived with disability for 328 diseases and injuries for 195 countries, 1990–2016: a systematic analysis for the Global Burden of Disease Study 2016. Lancet.

[2] Afshin A, Forouzanfar MH, Reitsma MB, Sur P, Estep K, Lee A (2017). Health effects of overweight and obesity in 195 countries over 25 years. N Engl J Med.

[3] Ng M, Fleming T, Robinson M, Thomson B, Graetz N, Margono C (2014). Global, regional, and national prevalence of overweight and obesity in children and adults during 1980-2013: a systematic analysis for the Global Burden of Disease Study 2013. Lancet..

[4] Poirier P, Giles TD, Bray GA, Hong Y, Stern JS, Pi-Sunyer FX (2006). Obesity and cardiovascular disease: pathophysiology, evaluation, and effect of weight loss: an update of the 1997 American Heart Association Scientific Statement on Obesity and Heart Disease from the Obesity Committee of the Council on Nutrition, Physical Activity, and Metabolism. Circulation..

[5] Okosun IS (2000). Ethnic differences in the risk of type 2 diabetes attributable to differences in abdominal adiposity in American women. J Cardiovasc Risk.

[6] Okosun IS, Boltri JM, Anochie LK, Chandra KM (2004). Racial/ethnic differences in prehypertension in American adults: population and relative attributable risks of abdominal obesity. J Hum Hypertens.

[7] Okosun IS, Boltri JM, Hepburn VA, Eriksen MP, Davis-Smith M (2006). Regional fat localizations and racial/ethnic variations in odds of hypertension in at-risk American adults. J Hum Hypertens.

[8] Huxley R, Mendis S, Zheleznyakov E, Reddy S, Chan J (2010). Body mass index, waist circumference and waist:hip ratio as predictors of cardiovascular risk--a review of the literature. Eur J Clin Nutr.

[9] Huxley R, James WP, Barzi F, Patel JV, Lear SA, Suriyawongpaisal P (2008). Ethnic comparisons of the cross-sectional relationships between measures of body size with diabetes and hypertension. Obes Rev.

[10] Lee CM, Huxley RR, Wildman RP, Woodward M (2008). Indices of abdominal obesity are better discriminators of cardiovascular risk factors than BMI: a meta-analysis. J Clin Epidemiol.

[11] World Health Organization (2008). Waist circumference and waist-hip ratio: report of a WHO expert consultation.

[12] Ramírez-Vélez R, Pérez-Sousa M, Izquierdo M, Cano-Gutierrez CA, González-Jiménez E, Schmidt-RioValle J, et al. Validation of surrogate anthropometric indices in older adults: what is the best indicator of high cardiometabolic risk factor clustering? Nutrients. 2019;11(8):1701. 10.3390/nu11081701 PMC6723899 31344803

[13] Krakauer NY, Krakauer JC (2018). Untangling waist circumference and hip circumference from body mass index with a body shape index, hip index, and anthropometric risk indicator. Metab Syndr Relat Disord.

[14] Ricalde A, Allison M, Rifkin D, Shaw R (2018). Anthropometric measures of obesity and renal artery calcification: results from the multi-ethnic study of atherosclerosis. Atherosclerosis..

[15] Kerkadi A, Suleman D, Abu Salah L, Lotfy C, Attieh G, Bawadi H (2020). Adiposity indicators as cardio-metabolic risk predictors in adults from country with high burden of obesity. Diabetes Metab Syndr Obes.

[16] Pan American Health Organization. Economic dimensions of noncommunicable diseases in Latin America and the Caribbean: World Health Organization; 2016. Online: https://iris.paho.org/bitstream/handle/10665.2/28501/9789275119051_eng.pdf?sequence=1&isAllowed=y.

[17] Pan American Health Organization (2015). Profile of capacity and response to non-communicable diseases and their risk factors in the region of the Americas.

[18] Rodriguez CJ, Allison M, Daviglus ML, Isasi CR, Keller C, Leira EC (2014). Status of cardiovascular disease and stroke in Hispanics/Latinos in the United States: a science advisory from the American Heart Association. Circulation..

[19] Mozaffarian D, Benjamin Emelia J, Go Alan S, Arnett Donna K, Blaha Michael J, Cushman M (2016). Heart disease and stroke statistics—2016 update. Circulation..

[20] Pool Lindsay R, Ning H, Lloyd-Jones Donald M, Allen NB. Trends in racial/ethnic disparities in cardiovascular health among US adults from 1999–2012. J Am Heart Assoc. 2017;6(9):e006027. 10.1161/JAHA.117.006027 PMC5634269 28939713

[21] Maharaj R, Thompson T, Nunez M, Nazario C, Adams O, Nunes P, et al. The Eastern Caribbean Health Outcomes Research Network. Caribb Med J. 2012;74(2):36.

[22] Wang KH, Thompson TA, Galusha D, Friedman H, Nazario CM, Nunez M (2018). Non-communicable chronic diseases and timely breast cancer screening among women of the Eastern Caribbean Health Outcomes Research Network (ECHORN) Cohort Study. Cancer Causes Control.

[23] Hassan S, Ojo T, Galusha D, Martinez-Brockman JL, Adams OP, Maharaj R, et al. Obesity and weight misperception among adults in the Eastern Caribbean Health Outcomes Research Network (ECHORN) Cohort Study. Obes Sci Pract. 2018;4(4):367–78. 10.1002/osp4.280 PMC6105698 30151231

[24] Oladele CR, Thompson TA, Wang K, Galusha D, Tran E, Martinez-Brockman JL (2020). Egocentric health networks and cardiovascular risk factors in the ECHORN cohort study. J Gen Intern Med.

[25] ECHORN Coordinating Center. ECHORN Cohort Study Baseline Survey. ecc@yale.edu. 2013.

[26] Goff David C, Lloyd-Jones Donald M, Bennett G, Coady S, D’Agostino Ralph B, Gibbons R (2014). 2013 ACC/AHA guideline on the assessment of cardiovascular risk. Circulation.

[27] Whelton PK, Carey RM, Aronow WS, Casey DE, Collins KJ, Dennison Himmelfarb C (2018). 2017 ACC/AHA/AAPA/ABC/ACPM/AGS/APhA/ASH/ASPC/NMA/PCNA guideline for the prevention, detection, evaluation, and management of high blood pressure in adults. J Am Coll Cardiol.

[28] Stone NJ, Robinson JG, Lichtenstein AH, Bairey Merz CN, Blum CB, Eckel RH (2014). 2013 ACC/AHA guideline on the treatment of blood cholesterol to reduce atherosclerotic cardiovascular risk in adults: a report of the American College of Cardiology/American Heart Association Task Force on Practice Guidelines. Circulation..

[29] Grundy Scott M, Stone Neil J, Bailey Alison L, Beam C, Birtcher Kim K, Blumenthal Roger S (2019). 2018 AHA/ACC/AACVPR/AAPA/ABC/ACPM/ADA/AGS/APhA/ASPC/NLA/PCNA guideline on the management of blood cholesterol: a report of the American College of Cardiology/American Heart Association Task Force on Clinical Practice Guidelines. Circulation..

[30] Bibbins-Domingo K (2016). Aspirin use for the primary prevention of cardiovascular disease and colorectal cancer: U.S. preventive services task force recommendation statement. Ann Intern Med.

[31] Thompson FE, Midthune D, Kahle L, Dodd KW (2017). Development and evaluation of the National Cancer Institute’s dietary screener questionnaire scoring algorithms. J Nutr.

[32] National Cancer Institute. Dietary Screenr Questionnaires (DSQ) in the NHANES 2009-2010: SAS Programs National Cancer Institute,2018 [updated Feb 13, 2018. Available from: https://epi.grants.cancer.gov/nhanes/dietscreen/programs.html.

[33] Leening MJ, Vedder MM, Witteman JC, Pencina MJ, Steyerberg EW (2014). Net reclassification improvement: computation, interpretation, and controversies: a literature review and clinician's guide. Ann Intern Med.

[34] Okosun IS, Cooper RS, Rotimi CN, Osotimehin B, Forrester T (1998). Association of waist circumference with risk of hypertension and type 2 diabetes in Nigerians, Jamaicans, and African-Americans. Diabetes Care.

[35] Tarleton HP, Smith LV, Zhang Z-F, Kuo T (2014). Utility of anthropometric measures in a multiethnic population: their association with prevalent diabetes, hypertension and other chronic disease comorbidities. J Community Health.

[36] Qi Q, Strizich G, Hanna DB, Giacinto RE, Castaneda SF, Sotres-Alvarez D (2015). Comparing measures of overall and central obesity in relation to cardiometabolic risk factors among US Hispanic/Latino adults. Obesity (Silver Spring).

[37] Price GM, Uauy R, Breeze E, Bulpitt CJ, Fletcher AE (2006). Weight, shape, and mortality risk in older persons: elevated waist-hip ratio, not high body mass index, is associated with a greater risk of death. Am J Clin Nutr.

[38] Sargeant LA, Bennett FI, Forrester TE, Cooper RS, Wilks RJ (2002). Predicting incident diabetes in Jamaica: the role of anthropometry. Obes Res.

[39] Barbosa AR, Munaretti DB, Da Silva CR, Borgatto AF (2011). Anthropometric indexes of obesity and hypertension in elderly from Cuba and Barbados. J Nutr Health Aging.

[40] Nemesure B, Wu SY, Hennis A, Leske MC (2008). The relationship of body mass index and waist-hip ratio on the 9-year incidence of diabetes and hypertension in a predominantly African-origin population. Ann Epidemiol.

[41] Ramsaran C, Maharaj RG. Normal weight obesity among young adults in Trinidad and Tobago: prevalence and associated factors. Int J Adolesc Med Health. 2017;29(2). 10.1515/ijamh-2015-0042 26556836

[42] Motamed N, Perumal D, Zamani F, Ashrafi H, Haghjoo M, Saeedian FS (2015). Conicity index and waist-to-hip ratio are superior obesity indices in predicting 10-year cardiovascular risk among men and women. Clin Cardiol.

[43] Al-Lawati JA, Barakat NM, Al-Lawati AM, Mohammed AJ (2008). Optimal cut-points for body mass index, waist circumference and waist-to-hip ratio using the Framingham coronary heart disease risk score in an Arab population of the Middle East. Diab Vasc Dis Res.

[44] Palacios C, Pérez CM, Guzmán M, Ortiz AP, Ayala A, Suárez E (2011). Association between adiposity indices and cardiometabolic risk factors among adults living in Puerto Rico. Public Health Nutr.

[45] Myint PK, Kwok CS, Luben RN, Wareham NJ, Khaw KT (2014). Body fat percentage, body mass index and waist-to-hip ratio as predictors of mortality and cardiovascular disease. Heart..

[46] Bell RA, Chen H, Saldana S, Bertoni AG, Effoe VS, Hairston KG (2018). Comparison of measures of adiposity and cardiovascular disease risk factors among African American adults: the Jackson Heart Study. J Racial Ethn Health Disparities.

[47] Ashwell M, Gunn P, Gibson S (2012). Waist-to-height ratio is a better screening tool than waist circumference and BMI for adult cardiometabolic risk factors: systematic review and meta-analysis. Obes Rev.

[48] Daviglus ML, Talavera GA, Avilés-Santa M (2012). Prevalence of major cardiovascular risk factors and cardiovascular diseases among hispanic/latino individuals of diverse backgrounds in the United States. JAMA..

[49] Hales CMCM, Fryar CD, Ogden CL (2017). Prevalence of obesity among adults and youth: United States, 2015–2016.

[50] Kavousi M, Leening MG, Nanchen D (2014). Comparison of application of the acc/aha guidelines, adult treatment panel iii guidelines, and european society of cardiology guidelines for cardiovascular disease prevention in a european cohort. JAMA..

[51] DeFilippis AP, Young R, Carrubba CJ, McEvoy JW, Budoff MJ, Blumenthal RS (2015). An analysis of calibration and discrimination among multiple cardiovascular risk scores in a modern multiethnic cohort. Ann Intern Med.

[52] Rana JS, Tabada GH, Solomon MD, Lo JC, Jaffe MG, Sung SH (2016). Accuracy of the atherosclerotic cardiovascular risk equation in a large contemporary, multiethnic population. J Am Coll Cardiol.

[53] Fox ER, Samdarshi TE, Musani SK, Pencina MJ, Sung JH, Bertoni AG (2016). Development and validation of risk prediction models for cardiovascular events in black adults: the Jackson heart study cohort. JAMA Cardiol.

[54] Romero-Corral A, Somers VK, Sierra-Johnson J, Korenfeld Y, Boarin S, Korinek J (2010). Normal weight obesity: a risk factor for cardiometabolic dysregulation and cardiovascular mortality. Eur Heart J.

