# Supplementary material for: Anthropometric measures of obesity and associated cardiovascular disease risk in the Eastern Caribbean Health Outcomes Research Network (ECHORN) Cohort Study
Source: BMC Public Health. 2021 Feb 25;21:399. doi: 10.1186/s12889-021-10399-3 (PMC7905572; doi:10.1186/s12889-021-10399-3)
Supplement: Supplementary file 1 — Additional file 1: Appendices. Appendix A. Comparison of included and excluded cases from the ECS. Appendix B. Odds of elevated cardiovascular risk by elevated BMI, waist-to-hip ratio and waist-to-height ratio stratified by race. Appendix C. Odds of elevated cardiovascular risk by elevated BMI, waist-to-hip ratio and waist-to-height ratio [all participants classified as “AFRICAN AMERICAN” except those who self-identify as white – classified as “OTHER” for ASCVD Calculation]. [file 12889_2021_10399_MOESM1_ESM.docx]

|  | Study Sample (1,617) | | Excluded Cases (n=1,344) | | p-value |
| --- | --- | --- | --- | --- | --- |
|  | **n** | **%** | **n** | **%** |  |
| **Age** |  |  |  |  | 0.0002 |
| 40-49 | 442 | 27.3 | 300 | 22.3 |  |
| 50-59 | 588 | 36.4 | 457 | 34.0 |  |
| 60-69 | 397 | 24.6 | 379 | 28.2 |  |
| 70+ | 190 | 11.8 | 208 | 15.5 |  |
| **Gender** |  |  |  |  | 0.2262 |
| Male | 577 | 35.7 | 451 | 33.6 |  |
| Female | 1040 | 64.3 | 893 | 66.4 |  |
| **Race** |  |  |  |  | <.0001 |
| Black/Caribbean | 828 | 51.2 | 718 | 66.0 |  |
| East Indian | 124 | 7.7 | 95 | 8.7 |  |
| Hispanic/PR | 335 | 20.7 | 102 | 9.4 |  |
| Mixed | 195 | 12.1 | 118 | 10.9 |  |
| Other | 3 | 0.2 | 3 | 0.3 |  |
| White | 132 | 8.2 | 52 | 4.8 |  |
| **Elevated BMI** |  |  |  |  | 0.2388 |
| not elevated | 1004 | 62.1 | 783 | 60.0 |  |
| elevated | 613 | 37.9 | 523 | 40.1 |  |
| **Waist-to-hip Ratio** |  |  |  |  | 0.3557 |
| not elevated | 803 | 49.7 | 630 | 48.0 |  |
| elevated | 814 | 50.3 | 684 | 52.1 |  |
| **Waist Circumference** |  |  |  |  | 0.908 |
| not elevated | 842 | 52.1 | 684 | 51.9 |  |
| elevated | 775 | 47.9 | 635 | 48.1 |  |
| **Waist to height ratio** |  |  |  |  | 0.7666 |
| not elevated | 338 | 20.9 | 281 | 21.4 |  |
| elevated | 1279 | 79.1 | 1035 | 78.7 |  |
| **Location** |  |  |  |  | <.0001 |
| BB | 432 | 26.72 | 576 | 42.9 |  |
| PR | 599 | 37.04 | 172 | 12.8 |  |
| TT | 439 | 27.15 | 390 | 29.0 |  |
| USVI | 147 | 9.09 | 206 | 15.3 |  |

**APPENDIX A:** Comparison of included and excluded cases from the ECS

Values are n| (col %) for categorical variables

**APPENDIX B:** Odds of elevated cardiovascular risk by elevated BMI, waist-to-hip ratio and waist-to-height ratio stratified by race

| Anthropometric Measure | **Black/Caribbean** | | | | | |
| --- | --- | --- | --- | --- | --- | --- |
|  | Odds of CVD Risk > 7.5% | | Odds of CVD Risk > 10% | | Odds of CVD Risk > 20% | |
|  | OR (95% CI) | p-value | OR (95% CI) | p-value | OR (95% CI) | p-value |
| Elevated BMI | 0.85 (0.63-1.15) | 0.2896 | 0.97 (0.71-1.33) | 0.8604 | 1.15 (0.75-1.75) | 0.5268 |
| Elevated Waist to Hip Ratio | 2.20 (1.65-2.94) | <.0001 | 2.40 (1.77-3.27) | <.0001 | 3.08 (1.98-4.8) | <.0001 |
| Elevated Waist to Height Ratio | 1.63 (1.16-2.28) | 0.0051 | 1.80 (1.24-2.62) | 0.0019 | 1.73 (1-2.99) | 0.0497 |

| Anthropometric Measure | **Other** | | | | | |
| --- | --- | --- | --- | --- | --- | --- |
|  | Odds of CVD Risk > 7.5% | | Odds of CVD Risk > 10% | | Odds of CVD Risk > 20% | |
|  | OR (95% CI) | p-value | OR (95% CI) | p-value | OR (95% CI) | p-value |
| Elevated BMI | 0.56 (0.39-0.8) | 0.0014 | 0.52 (0.34-0.78) | 0.002 | 0.48 (0.25-0.93) | 0.0285 |
| Elevated Waist to Hip Ratio | 3.62 (2.48-5.29) | <.0001 | 4.01 (2.55-6.3) | <.0001 | 5.31 (2.45-11.51) | <.0001 |
| Elevated Waist to Height Ratio | 1.53 (0.92-2.53) | 0.1007 | 1.71 (0.94-3.12) | 0.0796 | 1.78 (0.69-4.63) | 0.2368 |

Adjusted multivariate regression analysis showing odds ratio for designated 10-year cardiovascular risk score category by anthropometric measure. Adjusted for educational level, added sugar intake, fruit/salad intake and physical activity level. CVD=Cardiovascular disease; OR=odds ratio; BMI=Body Mass Index; WHR=Waist-to-hip ratio; WHtR=waist-to-height ratio. Elevated BMI defined as ≥30kg/m^2^; elevated WHR defined as >0.9 (men), >0.85 (women); elevated WHtR defined as >0.5

**APPENDIX C:** Odds of elevated cardiovascular risk by elevated BMI, waist-to-hip ratio and waist-to-height ratio [all participants classified as “AFRICAN AMERICAN” except those who self-identify as white – classified as “OTHER” for ASCVD Calculation]

| Anthropometric Measure | **TOTAL** | | | | | |
| --- | --- | --- | --- | --- | --- | --- |
|  | Odds of CVD Risk > 7.5% | | Odds of CVD Risk > 10% | | Odds of CVD Risk > 20% | |
|  | OR (95% CI) | p-value | OR (95% CI) | p-value | OR (95% CI) | p-value |
| Elevated BMI | 0.83 (0.67,1.02) | 0.0825 | 0.93 (0.74,1.16) | 0.5161 | 0.93 (0.68,1.28) | 0.6656 |
| Elevated Waist to Hip Ratio | 2.46 (2.00,3.03) | <.0001 | 2.72 (2.17,3.41) | <.0001 | 4.02 (2.81,5.76) | <.0001 |
| Elevated Waist to Height Ratio | 1.71 (1.32,2.23) | <.0001 | 1.89 (1.41,2.54) | <.0001 | 2.02 (1.28,3.2) | 0.0027 |

| Anthropometric Measure | **MEN** | | | | | |
| --- | --- | --- | --- | --- | --- | --- |
|  | Odds of CVD Risk > 7.5% | | Odds of CVD Risk > 10% | | Odds of CVD Risk > 20% | |
|  | OR (95% CI) | p-value | OR (95% CI) | p-value | OR (95% CI) | p-value |
| Elevated BMI | 1.11 (0.76,1.61) | 0.5823 | 1.21 (0.83,1.74) | 0.3226 | 1.23 (0.78,1.93) | 0.3816 |
| Elevated Waist to Hip Ratio | 2.75 (1.94,3.9) | <.0001 | 3.23 (2.26,4.62) | <.0001 | 4.19 (2.53,6.92) | <.0001 |
| Elevated Waist to Height Ratio | 1.98 (1.35,2.91) | 0.0005 | 2.34 (1.56,3.51) | <.0001 | 3.52 (1.91,6.51) | <.0001 |

| Anthropometric Measure | **WOMEN** | | | | | |
| --- | --- | --- | --- | --- | --- | --- |
|  | Odds of CVD Risk > 7.5% | | Odds of CVD Risk > 10% | | Odds of CVD Risk > 20% | |
|  | OR (95% CI) | p-value | OR (95% CI) | p-value | OR (95% CI) | p-value |
| Elevated BMI | 0.91 (0.69,1.21) | 0.5176 | 1.03 (0.76,1.39) | 0.8677 | 1.12 (0.69,1.81) | 0.6603 |
| Elevated Waist to Hip Ratio | 2.26 (1.71,2.99) | <.0001 | 2.32 (1.71,3.16) | <.0001 | 3.55 (2.06,6.11) | <.0001 |
| Elevated Waist to Height Ratio | 2.49 (1.62,3.83) | <.0001 | 2.36 (1.46,3.81) | 0.0005 | 1.57 (0.75,3.25) | 0.2298 |

Adjusted multivariate regression analysis showing odds ratio for designated 10-year cardiovascular risk score category by anthropometric measure. Adjusted for educational level, added sugar intake, fruit/salad intake and physical activity level. CVD=Cardiovascular disease; OR=odds ratio; BMI=Body Mass Index; WHR=Waist-to-hip ratio; WHtR=waist-to-height ratio. Elevated BMI defined as ≥30kg/m^2^; elevated WHR defined as >0.9 (men), >0.85 (women); elevated WHtR defined as >0.5
